# Predictors of inhospital mortality and re-hospitalization in older adults with community-acquired pneumonia: a prospective cohort study

**DOI:** 10.1186/1471-2318-10-22

**Published:** 2010-05-11

**Authors:** Binod Neupane, Stephen D Walter, Paul Krueger, Tom Marrie, Mark Loeb

**Affiliations:** 1 Department of Clinical Epidemiology and Biostatistics, McMaster University, Hamilton, Ontario, Canada; 2 Department of Family and Community Medicine, University of Toronto, Toronto, Canada; 3 Dalhousie University, Halifax, Nova Scotia, Canada; 4 Departments of Pathology and Molecular Medicine, McMaster University, Hamilton, Ontario, Canada; 5 Michael DeGroote Institute for Infectious Diseases, McMaster University, Hamilton, Ontario, Canada

## Abstract

**Background:**

A better understanding of potentially modifiable predictors of in-hospital mortality and re-admission to the hospital following discharge may help to improve management of community-acquired pneumonia in older adults. We aimed to assess the associations of potentially modifiable factors with mortality and re-hospitalization in older adults hospitalized with community-acquired pneumonia.

**Methods:**

A prospective cohort study was conducted from July 2003 to April 2005 in two Canadian cities. Patients aged 65 years or older hospitalized for community-acquired pneumonia were followed up for up to 30 days from initial hospitalization for mortality and these patients who were discharged alive within 30 days of initial hospitalization were followed up to 90 days of initial hospitalization for re-hospitalization. Separate logistic regression analyses were performed identify the predictors of mortality and re-hospitalization.

**Results:**

Of 717 enrolled patients hospitalized for community-acquired pneumonia, 49 (6.8%) died within 30 days of hospital admission. Among these patients, 526 were discharged alive within 30 days of hospitalization of whom 58 (11.2%) were re-hospitalized within 90 days of initial hospitalization. History of hip fracture (odds ratio (OR) = 4.00, 95% confidence interval (CI) = (1.46, 10.96), P = .007), chronic obstructive pulmonary disease (OR = 2.31, 95% CI = (1.18, 4.50), P = .014), cerebrovascular disease (OR = 2.11, 95% CI = (1.03, 4.31), P = .040) were associated with mortality. Male sex (OR = 2.35, 95% CI = (1.13, 4.85), P = .022) was associated with re-hospitalization while vitamin E supplementation was protective (OR = 0.37 (0.16, 0.90), P = .028). Lower socioeconomic status, prior influenza and pneumococcal vaccinations, appropriate antibiotic prescription upon admission, and lower nutrition risk were not significantly associated with mortality or re-hospitalization.

**Conclusion:**

Chronic comorbidities appear to be the most important predictors of death and re-hospitalization in older adults hospitalized with community-acquired pneumonia while vitamin E supplementation was protective.

## Background

Community-acquired pneumonia (CAP) is one of the leading causes of mortality in older adults aged 65 years or more [1-6]. Re-hospitalization due to pneumonia after premature hospital discharge or due to new or worsening of co-existing medical complications is associated with extra use of medical resources, increased costs and deaths [7].

Demographic factors such as age and comorbidities such as chronic lung diseases (COPD) may not fully explain mortality and re-hospitalization associated with CAP in the elderly. A better understanding of potentially modifiable predictors of in-hospital mortality and re-admission to the hospital following discharge may help to improve management of pneumonia in this vulnerable population. However, conflicting evidence exists regarding the role of modifiable factors, such as influenza and pneumococcal vaccines [8-10] and guideline-based antimicrobial use [11-14]. Malnutrition has been found to be associated with mortality [15,16] and re-admission [15] of patients discharged alive within 30 days of initial hospitalization. Poor functional status has also been associated with 30-day in-hospital mortality [15,17]. However, this finding was not seen in another study [18]. Problems with these studies include small sample sizes [14-18], retrospective designs based on administrative records [11,12], and/or failure to use rigorous definition of CAP [18]. Accurate identification of pneumonia cases could be a problem especially in retrospective studies using administrative data (where up to 40% of all cases of pneumonia diagnosed by ER physicians were not confirmed as so by radiologists) [19].

In this prospective cohort study where all pneumonia cases were confirmed by radiologists, we sought to identify the predictors of 30-day mortality in older adults with CAP and hospital re-admission within 90 days among these patients who were discharged alive to home, nursing home or another hospital within 30 days of their initial hospitalization. We specifically sought to assess the associations of potentially modifiable risk factors including immunization history, empiric antimicrobial use, as well as socioeconomic, nutritional and functional statuses.

## Methods

### Study Participants

In this prospective cohort study, patients aged 65 years and older who were hospitalized for CAP in 10 hospitals in Hamilton, Ontario and Edmonton, Alberta, Canada were enrolled from 2003 to 2005. There are four emergency departments (EDs) in Hamilton, serving a catchment area of approximately 2.2 million people, and six EDs in Edmonton, serving a catchment area of about 1 million people. Eligible participants had to live in one of these two catchment area communities and have at least two of the following signs and symptoms: temperature >38°C, productive cough, chest pain, shortness of breath, or crackles on auscultation. They also had to have a new opacity on a chest radiograph that was interpreted by a radiologist as being compatible with pneumonia. Exclusion criteria were residing in a nursing home and infection at another site in addition to CAP.

The study was approved by the Hamilton Health Sciences Research Ethics Board, St. Joseph's Hospital Research Ethics Board, and the University of Alberta Research Ethics Board. Informed consent was obtained from all participants.

### Data

Trained interviewers collected health data of the initial hospital admission after the patients were identified in EDs and enrolled in the study. Demographic, comorbidity, lifestyle and other health related data were collected from patients by personal interviews using structured questionnaires and additional complementary information was extracted from patients' medical charts. The demographic variables included age, sex, height and weight to calculate body mass index, marital status (whether married and living with spouse or not), and living arrangement (alone or in a family). Comorbidity variables included asthma, cancer, chronic obstructive pulmonary disease (COPD), congestive heart failure, cerebrovascular disease, liver disease, renal disease, and diabetes mellitus. In addition, data was collected on mental confusion, vital signs, empiric antibiotics used upon admission, medication history including use of vitamins, minerals and immunosuppression drugs, and influenza and pneumococcal immunizations prior to hospital admission.

Level of education was used as a proxy for socioeconomic status since older adults may not have income from active employment. Patients were considered having low socioeconomic status if they had less than high school education. Life-style variables included smoking (whether or not a patient smoked 100 or more cigarettes in lifetime), household smoking (whether or not any member in the patient's family smoked), amount of alcohol consumption (mean grams of alcohol per month in the last 12 months). Nutritional status was assessed using a 15-item adaptation of the Nutrition Screening Initiative Level I 10 statement [20] and the Mini-Nutritional Assessment [21,22], with scores ranging from 0 (high nutritional risk) to 60 (no nutritional risk). Patient's functional status was assessed using 10-item modified Barthel Index [23], with scores ranging from 0 (full dependence) to 20 (complete independence). The data were highly skewed with most participants having high scores; we dichotomized the Barthel Index score into 17 or less for lower functional status (some dependency) versus 18 to 20 for higher functional status (slight to no dependency). Severity of pneumonia illness at presentation was assessed using the British Thoracic Society's severity of illness index CRB-65 [24]. The CRB-65 score ranges from 1 (not severe) to 4 (highly severe) for elderly population. This is a more practical index for assessing severity of pneumonia illness at presentation because it is easy and quick to assess the severity, where blood urea measurement is omitted from the CURB-65 scale [24]. Antibiotics prescribed on admission were defined as "appropriate" if they were respiratory fluroqualone alone or a combination of advance macrolide and beta-lactum antibiotics. This classification is based on Canadian guidelines for the management of CAP in an in-patient setting [25].

Patients were followed up for 30 days from initial hospitalization for their mortality. The patients discharged alive to home, nursing home or another hospital within 30 days of initial hospitalization were followed up to 90 days of initial hospitalization and their hospital re-admission was determined by telephone interview or review of medical charts.

### Statistical Analysis

For the statistical analysis, we defined the outcome "mortality" event as any death that occurred within 30 days of the initial hospital admission. We also did a sensitivity analysis where we alternatively defined "mortality" outcome as any deaths within 30 days compared to discharges alive within 30 days, while excluding patients who were still in the hospital more than 30 days after the initial admission. "Re-hospitalization" was defined as hospitalization for any reason within 90 days of the initial hospitalization among those patients who were discharged alive within 30 days of the initial hospitalization.

In the univariate analysis, we tested the crude associations of variables of interest using two-sided tests (Chi-squared tests for binary variables and t-tests for continuous variables) with the mortality and readmission outcomes. Association was expressed as odds ratio (OR) with corresponding 95% confidence interval (CI) for each binary variable and as mean difference with corresponding 95% CI for each continuous variable.

In the multivariable regression model, certain variables of interest (i.e. lower socioeconomic status, appropriate antibiotic prescription upon admission, influenza vaccination, pneumococcal vaccination, lower nutritional risk (i.e., higher nutrition score), and lower functional status) were selected *a priori *for adjustment whereas the other variables were considered as candidate variables for multivariable model of each outcome if they had p-value of ≤ .10 in the corresponding univariate analysis. We used logistic regression with a step-wise backward elimination approach to develop the final model for mortality and re-hospitalization separately. Thus, the final model for each outcome included the variables selected *a priori *and any other variables that remained statistically significant (p < 0.05) in two-sided tests. Adjusted associations are expressed as ORs and corresponding 95% CIs.

Data were analyzed using SAS for Windows version 9.1 (SAS Institute Inc. Cary, NC).

## Results

**Figure 1:**
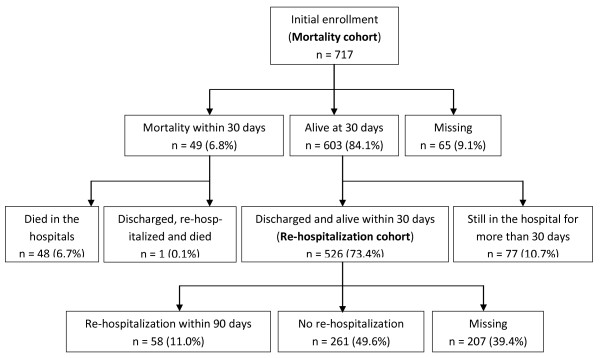
**Mortality and re-hospitalization in the cohort of patients hospitalized for community-acquired pneumonia**.

There were 717 patients (365 from Hamilton and 352 from Edmonton) enrolled. Forty nine (6.8%) died within 30 days and 603 (84.1%) were alive at 30 days of hospital admission of whom 526 (73.3%) were discharged alive from the hospitals to their homes, nursing homes or another hospitals and 77 (10.7%) were still in the same hospitals for more than 30 days (see Figure 1). Fifty eight (11.0%) of 526 patients discharged alive within 30 days of initial hospitalization were re-admitted within 90 days of initial hospitalization. Descriptive statistics by outcome status and unadjusted associations of factors with mortality and re-hospitalization are presented in Table 1 and Table 2, respectively. Adjusted associations with mortality and re-hospitalization are presented in Table 3 and 4, respectively.

**Table 1:** Descriptive statistics by mortality status and crude associations with mortality within 30 days of initial hospitalization for community acquired pneumonia.

Factors^a^	Mortality within 30 days (n = 49)	Alive at 30 days (n = 603)	Mortality vs. alive at 30 days	
			
			OR (95% CI)	P
**Demographic factors**				
Age (years)^b^	79.3 (8.6)	79.1 (7.6)	0.26 (-1.98, 2.50)	.819
Male Sex	31 (64.6)	365 (60.4)	1.19 (0.65, 2.21)	.571
Married, living with spouse	29 (60.4)	310 (51.8)	1.42 (0.78, 2.58)	.252
Living Alone	13 (26.5)	187 (32.6)	0.75 (0.39, 1.44)	.384
BMI^a^	24.7 (4.7)	25.4 (5.9)	-0.67 (-2.49, 1.15)	.471
**Low socioeconomic status**				
Education (< high school)	32 (65.3)	366 (61.5)	1.18 (0.64, 2.17)	.599
**Empiric Antibiotic Use**				
Appropriate Antibiotics	44 (89.8)	511 (83.9)	1.69 (0.65, 4.36)	.275
**Pneumonia Severity**				
CRB-65^a^	1.52 (0.7)	1.54 (0.7)	-0.02 (-0.21, 0.17)	.834
**Immunization history**				
Influenza Vaccine	38 (77.6)	473 (79.0)	0.92 (0.46, 1.85)	.816
Pneumococcal Vaccine	24 (49.0)	342 (57.8)	0.70 (0.39, 1.26)	.232
**Comorbidities**				
Asthma	13 (27.7)	142 (24.0)	1.21 (0.62, 2.35)	.576
Cancer	11 (22.9)	156 (26.3)	0.83 (0.41, 1.67)	.607
Cerebrovascular Disease^c^	16 (33.3)	115 (19.3)	2.08 (1.11, 3.93)	.021
Congestive Heart Failure	18 (39.1)	196 (33.5)	1.28 (0.69, 2.36)	.438
COPD^c^	31 (66.0)	284 (48.0)	2.10 (1.13, 3.92)	.018
Diabetes Mellitus	8 (16.7)	155 (26.0)	0.57 (0.26, 1.24)	.152
Dysphagia	10 (20.8)	78 (13.2)	1.73 (0.83, 3.62)	.138
Hip Fracture^c^	7 (14.6)	29 (4.9)	3.33 (1.38, 8.07)	.005
Liver Disease^c, d^	4 (8.7)	11 (1.9)	5.03 (1.54, 16.48)	.003
Renal Disease	9 (20.0)	109 (18.6)	1.10 (0.51, 2.34)	.812
**Mineral supplement and medication history**				
Vitamin E Supplementation	9 (18.3)	134 (23.1)	0.75 (0.35, 1.58)	.448
Zinc Supplementation	5 (10.2)	65 (11.4)	0.88 (0.34, 2.30)	.796
Immunosuppressive Drugs	8 (18.2)	93 (16.4)	1.13 (0.51, 2.51)	.760
**Lifestyle factors**				
Smoking (> 100 cigarettes)	39 (79.6)	447 (74.3)	1.35 (0.66, 2.77)	.409
Household Smoking	11 (22.5)	111 (18.4)	1.28 (0.63, 2.58)	.489
Alcohol (gram/month)^a^	16.1 (41.3)	10.6 (36.8)	5.46 (-5.48, 16.40)	.328
Nutritional Score^a^	45.3 (8.8)	47.0 (6.8)	-1.70 (-3.76, 0.35)	.198
**Low functional status**				
Barthel Score (≤ 17)	12 (24.5)	142 (23.7)	1.04 (0.53, 2.06)	.901

^a^Descriptive statistics are expressed as mean (standard deviation (SD)) for continuous variables and count (percentage) for binary variables

^b^Continuous variables

^c^p ≤ .10

^d^Rare exposure (not considered as candidate variable for multivariable analysis for mortality even when it was statistically significant in univariate analysis).

**Table 2:** Descriptive statistics by re-hospitalization status and crude associations with re-hospitalization with 90 days of initial hospitalization for community acquired pneumonia.

Factors^a^	Re-hospitalization within 90 days of initial hospitalization			
	
	Yes (n = 58)	No (n = 261)	OR (95% CI)	P
**Demographic factors**				
Age (years)^b^	79.3 (7.1)	79.8 (7.8)	-0.47 (-2.67, 1.72)	.673
Male Sex^c^	44 (75.9)	150 (58.1)	2.26 (1.18, 4.34)	.012
Married, living with spouse	33 (58.9)	131 (50.4)	1.41 (0.79, 2.54)	.246
Living Alone	13 (23.2)	84 (34.2)	0.58 (0.30, 1.14)	.114
BMI^a^	25.5 (6.0)	25.3 (5.6)	0.21 (-1.49, 1.91)	.810
**Low socioeconomic status**				
Education (< high school)^c^	45 (79.0)	158 (62.0)	2.30 (1.16, 4.57)	.015
**Empiric Antibiotics Use**				
Appropriate Antibiotics	50 (86.2)	216 (82.8)	1.30 (0.58, 2.93)	.523
**Pneumonia Severity**				
CRB-65^a^	1.5 (0.6)	1.56 (0.7)	-0.06 (-0.25, 0.13)	.555
**Immunization history**				
Influenza Vaccination	48 (82.8)	198 (78.0)	1.36 (0.65, 2.85)	.419
Pneumococcal Vaccination	33 (58.9)	142 (55.3)	1.16 (0.64, 2.09)	.616
**Comorbidities**				
Asthma	14 (25.5)	62 (24.5)	1.05 (0.54, 2.06)	.882
Cancer	17 (32.1)	66 (26.0)	1.35 (0.71, 2.55)	364
Cerebrovascular Disease	15 (26.8)	56 (21.9)	1.31 (0.67, 2.53)	427
Congestive Heart Failure	24 (43.6)	95 (37.9)	1.27 (0.70, 2.30)	425
COPD	32 (58.2)	131 (51.2)	1.33 (0.74, 2.39)	345
Diabetes Mellitus	14 (25.0)	66 (25.5)	0.97 (0.50, 1.90)	940
Dysphagia	10 (17.9)	38 (14.9)	1.24 (0.58, 2.67)	579
Hip Fracture	2 (3.58)	11 (4.28)	0.83 (0.18, 3.84)	810
Liver Disease	1 (1.8)	2 (0.8)	2.3 (0.20, 25.82)	488
Renal Disease	13 (23.2)	40 (15.8)	1.61 (0.79, 3.26)	184
**Mineral supplement and medication history**				
Vitamin E Supplementation^c^	8 (15.1)	73 (28.9)	0.44 (0.20, 0.97)	039
Zinc Supplementation	5 (9.6)	33 (13.4)	0.69 (0.26, 1.86)	461
Immunosuppressive Drugs	14 (25.0)	40 (16.1)	1.73 (0.87, 3.47)	117
**Lifestyle factors**				
Smoking (> 100 cigarettes)	44 (77.2)	201 (77.3)	0.99 (0.50, 1.97)	985
Household Smoking	12 (21.1)	47 (18.1)	1.21 (0.59, 2.46)	601
Alcohol (gram/month)^a, c^	5.8 (15.8)	10.6 (31.6)	-4.86 (-13.33, 3.61)	093
Nutritional Score^a^	48.7 (5.9)	47.3 (6.7)	1.41 (-0.49, 3.31)	145
**Low functional status**				
Barthel Score (≤ 17)	9 (15.8)	60 (23.2)	0.62 (0.29, 1.34)	222

^a^Descriptive statistics are expressed as mean (standard deviation (SD)) for continuous variables and count (percentage) for binary variables

^b^Continuous variables

^c^p ≤ .10

**Table 3:** Adjusted associations with mortality within 30 days of initial hospitalization for community acquired pneumonia.

Factors	Mortality^a^		Mortality^b^	
		
	OR (95% CI)	P	OR (95% CI)	P
Lower socioeconomic status (Education < high school)	0.99 (0.51, 1.92)	983	0.98 (0.50, 1.91)	953
Influenza vaccination	1.36 (0.57, 3.21)	488	1.21 (0.51, 2.89)	660
Pneumococcal vaccination	0.64 (0.32, 1.27)	203	0.66 (0.33, 1.32)	243
Appropriate antibiotic use	1.71 (0.65, 4.52)	281	1.84 (0.69, 4.91)	220
Nutrition score	0.85 (0.68, 1.06)^c^	145	0.85 (0.68, 1.06)^c^	142
Lower functional status (Barthel score ≤ 17)	0.72 (0.33, 1.60)	423	0.70 (0.31, 1.56)	381
Hip Fracture	4.00 (1.46, 10.96)	007	4.27 (1.50, 12.18)	007
COPD	2.31 (1.18, 4.50)	014	2.25 (1.15, 4.39)	018
Cerebrovascular disease	2.11 (1.03, 4.31)	040	2.30 (1.11, 4.76)	025

^a^Mortality within 30 days vs. alive at 30 days of hospital admission

^b^Mortality within 30 days vs. discharged alive within 30 days of hospital admission (excluding those who remained in the hospitals for more than 30 days)

^**c**^Odds ratio for 5 points increment in score

**Table 4:** Adjusted association with re-hospitalization within 90 days of initial hospitalization for CAP.

Factors	OR (95% CI)	P
Lower socioeconomic status (Education < high school)	1.98 (0.95, 4.13)	069
Influenza vaccination	0.92 (0.39, 2.18)	842
Pneumococcal vaccination	1.45 (0.70, 3.02)	319
Appropriate antibiotic use	1.44 (0.58, 3.57)	428
Nutrition score	1.17 (0.89, 1.54)^a^	254
Lower functional status (Barthel score ≤ 17)	0.70 (0.31, 1.61)	406
Male Sex	2.35 (1.13, 4.85)	022
Vitamin E supplement	0.37 (0.16, 0.90)	028

OR = Odds ratio; CI = Confidence interval

^a^Odds ratio for 5 points increment

In the multivariable model, history of hip fracture (OR = 4.00, 95% CI = (1.46, 10.96), P = .007), chronic obstructive pulmonary disease (OR = 2.31, 95% CI = (1.18, 4.50), P = .014), and cerebrovascular disease (OR = 2.11, 95% CI = (1.03, 4.31), P = .040) were significantly associated with mortality (Table 3). In the sensitivity analysis that excluded patients who remained in hospital for more than 30 days, essentially the same results were found.

Only male sex (OR = 2.35, 95% CI = (1.13, 4.85), P = .022) was significantly associated with re-hospitalization while vitamin E supplementation was protective (OR = 0.37 (0.16, 0.90), P = .028) (Table 4).

## Discussion

We found that in older adults hospitalized with pneumonia, there was an increased risk of inhospital mortality associated with hip fracture, chronic obstructive pulmonary disease, and cerebrovascular disease. These findings are consistent with previous reports [14,26-28]. Among patients discharged from hospital within first 30 days, being male was associated with hospital re-admission within 90 days of initial hospitalization.

Taking vitamin E supplements was protective of re-hospitalization in our study. Vitamin E inadequacy leads to increased prostaglandin (PG)E2 production by alveolar macrophages, a hypothesized mechanism through which vitamin E deficiency may suppress T-cell-mediated immunity [29]. However, the clinical effect on outcomes is not well defined, with some studies showing benefit in those with deficiencies and other studies showing no benefit [30]. The fact however that these results are based on self-reported use of Vitamin E is an important limitation.

Lower educational level was not significantly associated with re-hospitalization. This is in contrast to other reports where lower education status and lack of employment have been associated with re-hospitalization in poor urban areas [30,31]. Given the risk of re-hospitalization nearly twice as high in those with lower educational status where p-value was close to .05 and many variables were adjusted for sample size that was reduced for re-hospitalization due missing values, it is possible that our study did not have sufficient power to demonstrate such an association.

Neither influenza nor pneumococcal vaccine appeared to have any protective effect on mortality or re-hospitalization. In a study, Herzog et al. found influenza vaccination to be protective against mortality and readmission in older adults hospitalized with pneumonia [8]. However, there are conflicting reports for pneumococcal vaccination [9,10]. It should be noted, however, that one of the concerns using observational designs to determine the benefits associated with immunizations is that individuals who are healthier may be more likely to be immunized and this could be the reason for observed protective effects. Moreover, thelow rate of death at 30 days (6.8%) may have limited our ability to detect a significant protective effect of pneumococcal vaccine.

We found no significant association between nutritional status and mortality or re-hospitalization. In contrast, Vecchiarino et al. found a positive association of death and re-hospitalization with malnutrition, assessed on the basis of a low serum albumin or a reported unintentional weight loss [15]. Requelme et al. also found a significant association between nutrition and mortality [16]. There are several possible explanations for these differences. For example, we might not have sufficient mortality and re-hospitalization cases to detect such associations in this cohort study. In addition, our sample was fairly homogeneous in terms of nutrition risk with 84.2% having higher one-third nutrition scores (scores between 40 and 60). As a result, our measurement might not have adequately discriminated high risk and low risk patients in terms of mortality or re-hospitalization.

We also did not observe any association between functional status and mortality or re-hospitalization. Vecchiarino et al. [15] and Cabre et al. [17], on the other hand, found that poor functional status was associated with 30-day mortality. However, the association with mortality was not significant in Mody and Bradley study [18]. One possible explanation is that the Barthel Index was insufficiently discriminatory in our population where most of the patients had very high Barthel scores.

Previous data suggest that appropriate antibiotic prescription prior to hospital admission is associated with reduced mortality in patients with community-acquired pneumonia [12,13]. However, we did not find such an association. This may be because there were relatively few inappropriate prescriptions (only 15.6% of all antibiotic prescriptions); so those who died were mostly from initial antibiotic appropriately prescribed group. Also, we might not expect an association between appropriate antibiotic prescription and re-hospitalization because impact of appropriate antibiotic prescription would have attenuated prior to hospital discharge when patients' conditions have stabilized (absence of fever and severe respiratory distress). Therefore, complications during hospitalization or new or coexisting medical illnesses would have potentially greater impact on re-hospitalization. Alternatively, there might be also significant variation in the effectiveness of different types and generations of antibiotics that we termed 'appropriate' collectively, especially among more severely ill patients who are more likely to die or have more severe complications[31].

We found associations between traditional variables and outcomes. But, none of the variables that we tested as potentially modifiable variables appeared to be statistically significant. Strength of our multi-centered study is that we considered pure pneumonia cases confirmed by radiologists following strict selection criteria where all exposure information was obtained prospectively. We also adjusted many variables of interests or confounding variables in order to minimize bias in the results. However, this was also the limitation of the study in terms of statistical power of the study because we had only few mortality and re-hospitalization events and the variables we assessed as potentially modifiable factors for mortality or re-hospitalization were either very common or very rare exposures in our cohort. Also, the effective sample size used for re-hospitalization was very small and as a consequence factors such as socio-economic status were not statistically significant even when the estimate of association was quite large. This is because we could not confirm re-hospitalization status for many participants who might either have moved out from their residential address or could not be contacted for other reasons. Since we were interested only on the factors at baseline or at the time of hospitalization, we did not consider and adjust any independent predictor of outcomes during hospitalization such as any superinfection e.g., diarrhea; or any other new and serious complications e.g., respiratory failure; or receiving extra medical attention, e.g., ICU admission; or drug related adverse events, e.g., adverse reaction. Some of these conditions might have affected the outcomes [32-34] and are also related to other variables introducing confounding bias as happens in observational studies.

## Conclusion

Chronic comorbidities appear to be the most important predictors of death and re-hospitalization in older adults hospitalized with community-acquired pneumonia. Vitamin E supplementation also appeared to be protective from (re)hospitalization for any disease following discharge from hospitals for pneumonia infection. Other variables that we tested as potentially modifiable to the in-hospital mortality and rehospitalization did not appear to have any associations.

## Competing interests

The authors declare that they have no competing interests.

## Authors' contributions

BN performed the statistical analysis, interpreted the results and drafted the manuscript. SDW decided analysis approach and helped in interpretation and drafting of the manuscript. PK participated in analyzing the data, interpretation of the results and drafting of the manuscript. TM participated in the design of the study and interpretation of the results. ML conceived of the study, and designed and coordinated the study, decided its analysis approach and helped to interpret the results and to draft the manuscript. All authors read and approved the final manuscript.

## Pre-publication history

The pre-publication history for this paper can be accessed here:

http://www.biomedcentral.com/1471-2318/10/22/prepub
